# Adipose stem cells-released extracellular vesicles as a next-generation cargo delivery vehicles: a survey of minimal information implementation, mass production and functional modification

**DOI:** 10.1186/s13287-022-02849-5

**Published:** 2022-05-03

**Authors:** Jianguo Chen, Ruiquan Liu, Tianyu Huang, Hengyun Sun, Haiyue Jiang

**Affiliations:** Plastic Surgery Hospital, Chinese Academy of Medical Sciences and Peking Union Medical College, 33 Badachu Road, Shijingshan District, Beijing, 100144 People’s Republic of China

**Keywords:** Adipose stem cells, Extracellular vesicles, Functional modification, Mass production, Minimal information, MISEV2018 guidelines

## Abstract

**Objectives:**

To investigate current situation of minimal information implementation highlighted by minimal information for studies of extracellular vesicles 2018 (MISEV2018) guidelines, and explore technological advances towards mass production and functional modification in aesthetic, plastic and reconstructive surgery.

**Methods:**

Original articles on extracellular vesicles (EVs) of adipose stem cells (ASCs) were identified. Statistics upon minimal information for EVs research, such as species, cell types, culture conditions, conditioned media harvesting parameters, EVs isolation/storage/identification/quantification, functional uptake and working concentration, were analyzed.

**Results:**

The items of cell culture conditions such as passage number, seeding density, conditioned media harvesting time, functional uptake and working concentration were poorly documented, with a reporting percentage of 47.13%, 54.02%, 29.89%, 62.07% and 36.21%, respectively. However, there were some studies not reporting information of ASCs origin, culture medium, serum, EVs isolation methods, quantification and identification of EVs, accounting for 3.45%, 10.34%, 6.90%, 3.45%, 18.39% and 4.02%, respectively. Serum deprivation and trophic factors stimuli were attempted for EVs mass production. Several technological advances towards functional modification included hypoxia pre-condition, engineering EVs and controlled release. Presently, ASCs EVs have been applied in multiple fields, including diabetic/non-diabetic wound healing, angiogenesis, inflammation modulation, fat grafting, hair regeneration, antiaging, and healing and regeneration of cartilage/bone/peripheral nerve/tendon.

**Conclusion:**

Our results highlight normative reporting of ASCs EVs in functional studies to increase reliability and reproducibility of scientific publications. The advances towards mass production and functional modification of ASCs EVs are also recommended to enhance therapeutic effects.

## Introduction

Adipose stem cells (ASCs) isolated from adipose tissues have emerged as a promising therapy for the healing of multiple tissues, such as wound healing [1], fat grafting [2], skin rejuvenation [3], cartilage [4] and bone regeneration [5]. The paracrine effect of ASCs is partly attributed to the extracellular vesicles (EVs) secretion. As a cell-free therapy, stem cell-derived EVs-associated intercellular communication has been widely studied for promoting regeneration and reconstruction of multiple tissues such as tendon [6] and bone regeneration [7]. EVs is the generic term for several subtypes of particles naturally released from the native cells, such as “exosome”, “microparticle/microvesicle”, “ectosome”, “oncosome”, “apoptotic body” and many other names [8]. With a size of about 50–200 nm, exosome is a subset of endosome-origin small EVs, known as a heterogeneous mixture of microRNA-assembled, protein-decorated and lipid-bound nanoparticles [9–11]. The last decades has witnessed a dramatically increasing number of scientific publications on ASCs EVs, opening new frontiers for a next-generation drug delivery platform in ASCs-based regenerative [10].

In 2018, the “minimal information for studies of extracellular vesicles 2018 (MISEV2018) guidelines” has sensitized researchers to follow normative outlines when reporting extracellular vesicles-associated studies [8]. However, some of the current scientific publications associated with ASCs EVs poorly followed these guidelines to clearly report minimal information, involving passage number [12], the name of culture medium [13], the source of species and adipose tissue [14], ASCs seeding density [15], conditioned media collection time [16] and working concentration [17], which would affect reliability and reproducibility of published results especially in the face of skepticism by researchers outside EVs. When translating EVs-therapy to clinical and industrial practices, the primary hurdle is the low yield. Several strategies, such as serum deprivation [18] and precondition of platelet-derived growth factor (PDGF) [19] have been used to stimulate ASCs EVs release. Another hurdle is the unsatisfactory therapeutic effects. Sometimes functional modification for EVs is necessary to enhance therapeutic roles, including but not limited to precondition of PDGF [19], hypoxia stimulus [20] and genetically engineered EVs through cell transfection [21] or electroporation [22]. It seems to be the productivity paradox between the remarkable advances in EVs research and the relatively slow growth of productivity.

In the wake of these hurdles, we carry out a systematic survey of scientific publications on ASCs EVs. We will critically discuss the status quo of minimal information implementation. Besides, we will outline the current technological advances towards mass production and functional modification for the potential *off*-*on-shelf* alternative to cell therapy. We also list the functional roles of ASCs EVs in the fields of aesthetic, plastic and reconstructive surgery.

## Methods

### Search strategy

We performed a systematic search in the PubMed, EMBASE and Cochrane Library databases involving ASCs EVs, without restrictions of language, publication year and publication status. A search strategy was generated using the following terms: “adipose stem cells,” “adipose stem cells,” “exosome,” and “extracellular vesicles”. We also reviewed reference lists of eligible studies and relevant reviews for additional articles. Those reviews, letters, comments, abstracts and publications irrelevant to ASCs EVs were excluded.

### Study selection

Two authors (J.G.C. and T.Y.H.) independently reviewed titles and abstracts of identified records, and full texts of potentially useful studies were reviewed. We resolved any disagreements through discussion with another author (H.Y.J.), and based on consensus, included or excluded those studies that we have discussed. The study was organized based on investigation of the minimal information and functional roles in aesthetic, plastic and reconstructive surgery.

## Results

### Search results

**Fig. 1:**
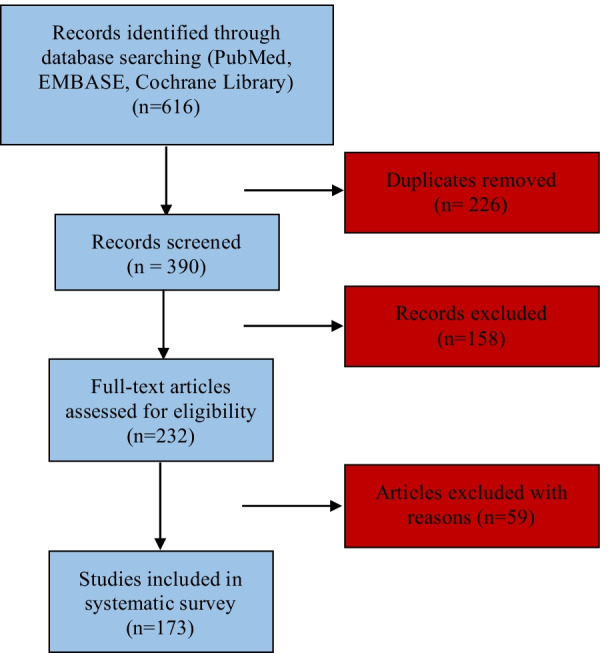
Flowchart of study search and selection

A total of 173 pre-clinical and clinical studies [11–183] between 2011 to 2021 were included for the statistical analysis of minimal information implementation, mass production, functional modification and functional roles in aesthetic, plastic and reconstructive surgery. The screening process is shown in Fig. 1.

### ASCs culture parameters

**Fig. 2:**
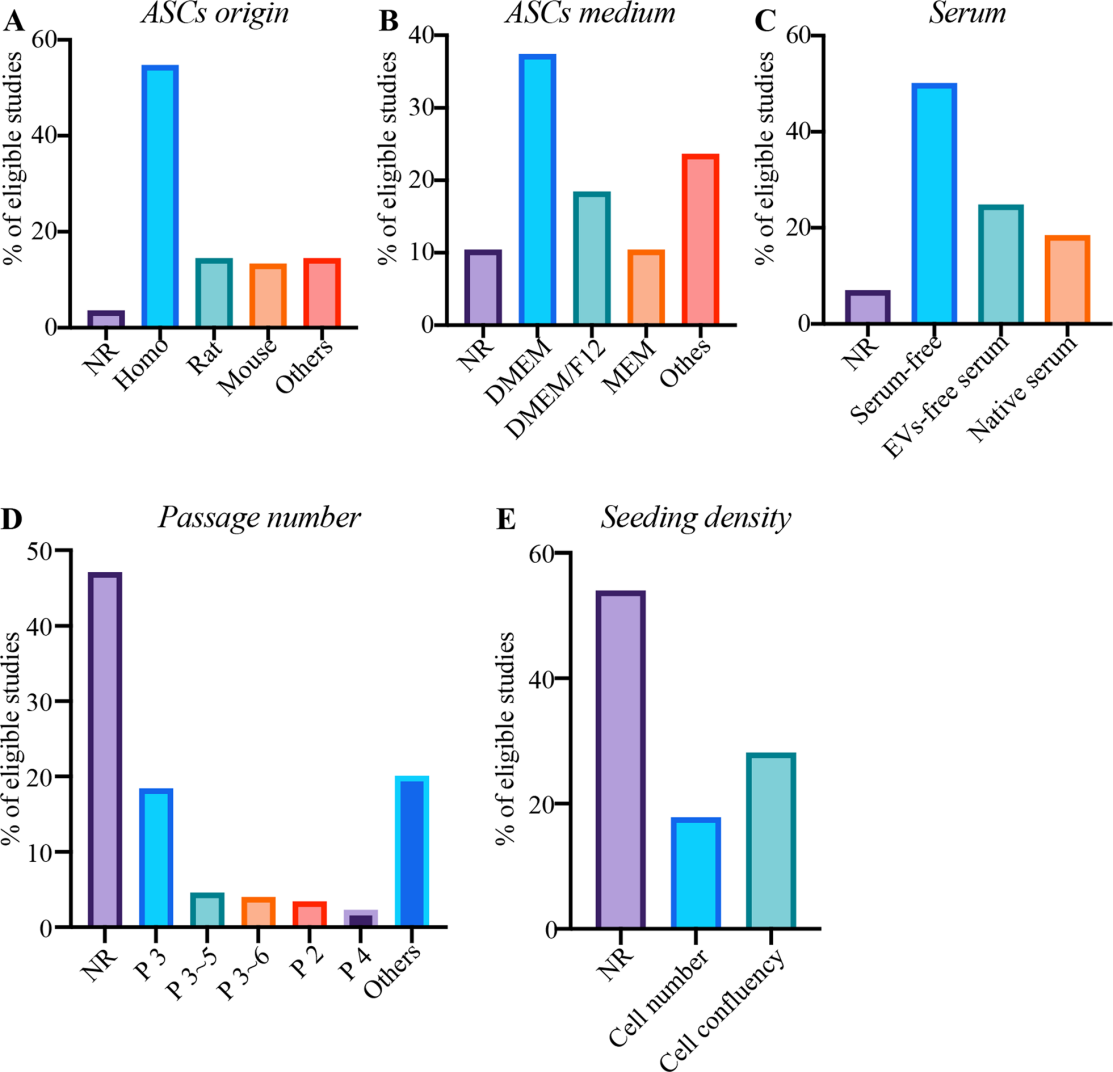
The percentage (%) of minimal information for **A** ASCs origin, **B** ASCs medium, **C** serum, **D** passage number, and **E** seeding density. *NR* percentage of “not reporting” minimal information

There were 3.45% of studies not reporting ASCs origin. The top three reporting derived types of ASCs were “homo, rat and mouse” adipose tissues (Fig. 2A). There were 10.34% of studies not clearly reporting the types of ASCs culture medium. The top three types were DMEM, DMEM/F-12 and MEM (Fig. 2B). Of the studies reporting DMEM, only 20.00% disclosed the use of high-glucose or low-glucose. 6.90% of studies did not document the use of serum for harvesting conditioned medium. 50.00% of studies used serum-free medium or serum replacement medium while 24.71% used EVs-depleted serum. However, the remaining studies used native serum without process of EVs-depletion (Fig. 2C). Almost half of studies did not document the passage number for EVs isolation. The top five reporting passage number were passage 3, passage 3 to 5, passage 3 to 6, passage 2 and passage 4 (Fig. 2D). Notably, there were more than half of studies not reporting ASCs seeding density. 28.16% of studies preferred to report degree of ASCs confluency as seeding density. (Fig. 2E).

### Technological advances towards mass production

**Fig. 3:**
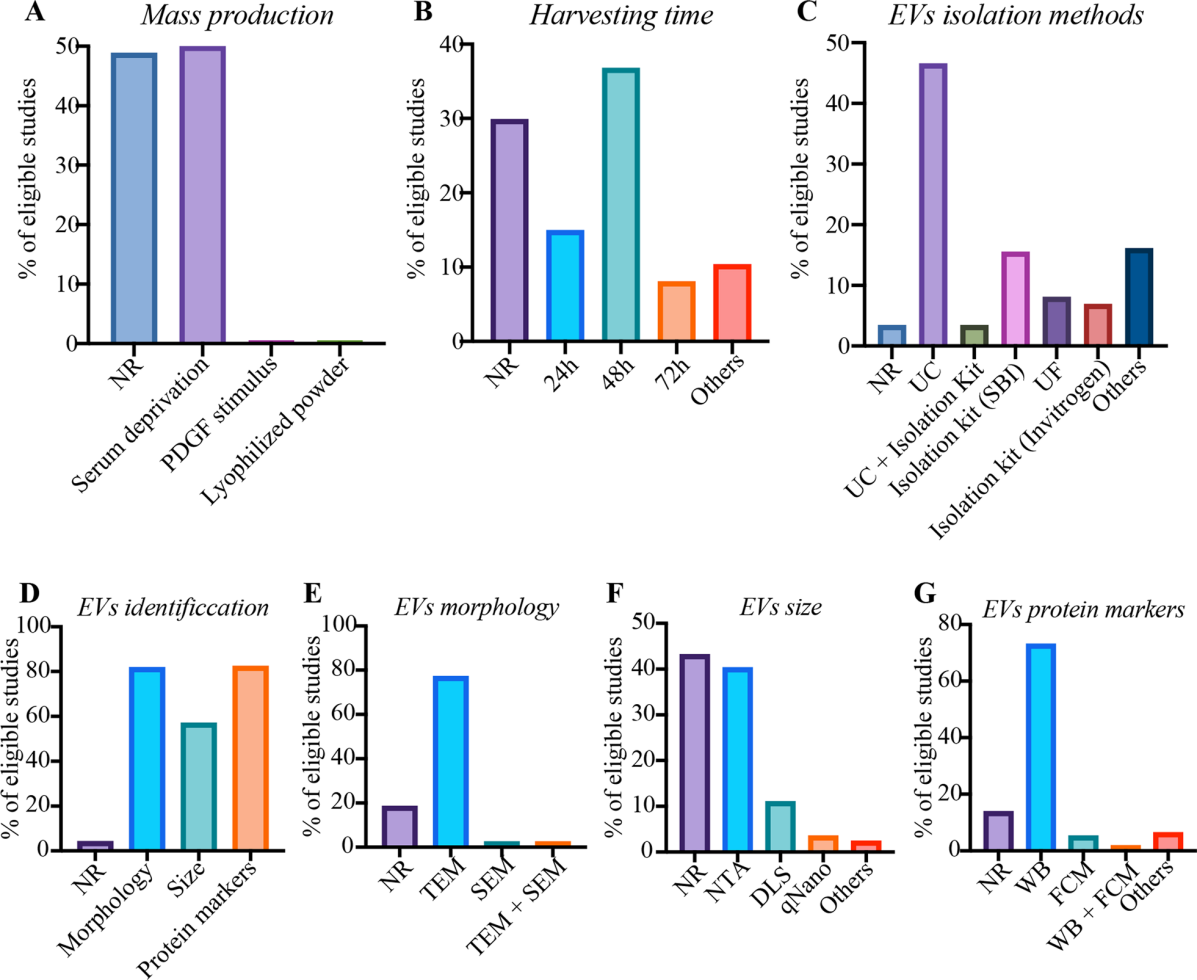
The percentage (%) of minimal information for **A** mass production, **B** conditioned medium harvesting time, **C** EVs isolation methods, **D** EVs identification, **E** EVs morphology, **F** EVs size distribution, and **G** EVs protein markers. NR: percentage of “not reporting” minimal information. *PDGF* platelet-derived growth factor, *UC* ultracentrifugation, *UF* ultrafiltration, *TEM* transmission electron microscope, *SEM* scanning electron microscope, *NTA* nanoparticle tracking analysis, *DLS* dynamic light scattering, *WB* western blotting, *FCM* flow cytometry

All included studies performed 2D-cell culture platforms for EVs production, without reporting use of hyperflasks, roller bottles, or 3D culture methods (e.g. perfusion, fixed bed or spinner flasks). Several physical or chemical stimulation was tried in 51.15% of studies to optimize EVs production. Serum deprivation was mostly used, accounting for 50.00%. Only one study reported precondition of ASCs with platelet-derived growth factor (PDGF). A study has evidenced that ASCs EVs could be stored in the form of lyophilized powder that could be helpful for stable storage and subsequent large scale production. There were no studies reporting methods of low pH, heat shock, glucose deprivation, ethanol, or ultrasounds for mass production. (Fig. 3A).

### Conditioned media harvesting parameters

29.89% of studies did not report the conditioned media harvesting time, but some of them document harvest in a cell confluence of 70% to 90%. The top three harvesting time were 48-h, 24-h and 72-h (Fig. 3B). Almost all studies chose to store the conditioned media at – 80 °C, or firstly isolated EVs and then stored it at – 80 °C.

### EVs isolation

There were 3.45% of studies not reporting EVs isolation methods. The top five isolation techniques were differential ultracentrifugation (UC), ExoQuick-TC reagent from *System Biosciences (SBI)*, ultrafiltration (UF), total EVs isolation kit from *Invitrogen, and UC plus* isolation kit (Fig. 3C).

### EVs identification

There were 4.02% of studies not reporting the information of EVs identification. The reporting percentages in terms of morphology, size distribution and protein markers were 81.61%, 56.90%, and 82.18%, respectively (Fig. 3D). Transmission electron microscope (TEM) was mostly used for detecting morphology (Fig. 3E). The top three size assessment tools were nanoparticle tracking analysis (NTA), dynamic light scattering (DLS) and qNano devices (Fig. 3F). Protein markers were mostly identified by western blotting. Particularly, the top five reporting markers were CD9, CD63, CD81, TSG101 and HSP70/90. Flow cytometry or flow cytometry combined with western blotting were also used for protein markers identification (Fig. 3G).

### Quantification, functional uptake through fluorescence labelling and working concentration of EVs

**Fig. 4:**
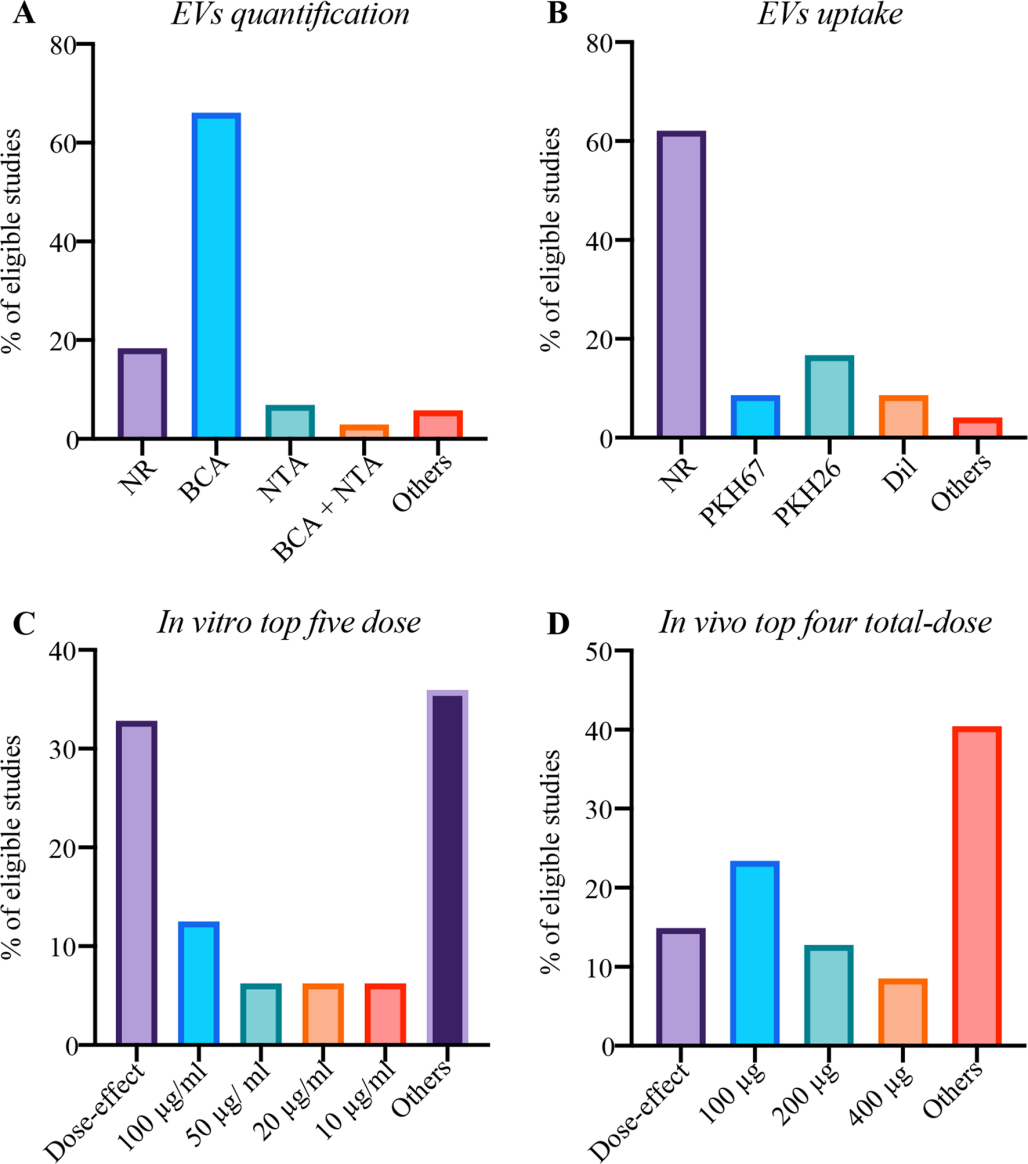
The percentage (%) of minimal information **A** EVs quantification, **B** EVs uptake, **C** in vitro top five dose, and **D** in vivo top four total-dose. *NR* percentage of “not reporting” minimal information, *BCA* bicinchoninic acid assay, *NTA* nanoparticle tracking analysis

There were 18.39% of studies not reporting quantification of EVs. Most of studies quantified EVs using BCA protein assay while only 6.90% of studies only using NTA. (Fig. 4A) There were 62.07% of studies not reporting functional uptake assays. The remaining studies mainly used PKH26, PKH67 and Dil as fluorescence labelling dyes (Fig. 4B). 36.21% of studies did not report working concentration in functional studies. The working concentration generally used in in vitro and in vivo studies are shown at Fig. 4C, D.

### The percentage of “not reporting” minimal information: before and after the publication of MISEV2018

We also conducted a comparison on the percentage of “not reporting” minimal information before and after the publication of MISEV2018. The results could be seen at Table 1. We found that the “not reporting” percentage of several parameters such as ASCs origin, isolation methods, EVs morphology/size/protein markers, EVs quantification and working concentration decreased to some extent after the publication of MISEV2018, indicating that MISEV2018 was favorable to promote the reporting of minimal information when performing ASCs EVs studies.

**Table 1:** The percentage (%) of “not reporting” minimal information before and after the publication of MISEV2018

Minimal information	Before (%)	After (%)	Comparison
ASCs origin	6.78	1.74	−
ASCs medium	10.17	10.43	+
Culture serum without EVs depletion/reporting	23.73	26.09	+
Harvesting time	28.81	30.43	+
Isolation methods	5.08	2.61	−
EVs morphology	30.51	12.17	−
EVs size	55.93	36.52	−
EVs protein markers	22.03	15.65	−
EVs quantification	27.12	13.91	−
EVs uptake	55.93	65.22	+
Working concentration	49.15	29.57	−

*ASCs* adipose stem cells, *EVs* extracellular vesicles

### Technological advances towards functional modification

THE modified strategies for enhancing loading and targeted delivery of EVs have been reported. Engineering EVs were mostly carried out either via transfecting functional molecules into ASCs [14, 17, 21, 26, 34, 35, 44, 45, 49, 72, 77, 79, 82, 113, 125, 181] or directly transfecting functional molecules into EVs [22, 37, 142]. Six studies [20, 67, 85, 86, 102, 103] reported the strategy of hypoxia culture precondition of ASCs. These hypoxia-preconditioned ASCs EVs shown superiority in RNA sequencing and functional assays such as fat grafting survival, neovascularization, inflammation inhibition, extracellular matrix regeneration, and pro-metabolism/pro-survival abilities.

Biomaterials laden with EVs were a promising strategy for controlled EVs release, which was especially helpful for chronic wound healing and bone regeneration [50, 88, 139, 140, 144, 145]. Seven kinds of regenerative biomaterials laden with EVs have been reported, including polypeptide-based FHE hydrogel, antioxidant polyurethane, hyaluronic acid, thermosensitive multifunctional polysaccharide-based dressing, alginate-based hydrogel, biohybrid bovine bone matrix and human acellular amniotic membrane.

Another strategy was the targeted differentiation induction. EVs released from osteogenic or chondrogenic induction of ASCs could specifically promote osteogenesis or chondrogenesis differentiation of MSCs [104, 151, 160, 182]. ASCs EVs could be modified in bone healing and regeneration via giving a stimulus of TNF-α or low-level laser irradiation (LLLI) to parent ASCs [114, 152]. The anti-inflammatory and immunosuppressive functions of ASCs EVs could be modified via giving an inflammatory stimulus of IFNγ and TNFα [92]. Stimulus of Platelet-derived growth factor (PDGF) could triggered the EVs secretion from parent ASCs and enhanced the angiogenic potential [19]. ASCs EVs from lean volunteers even were different from those from obese individuals in terms of protein markers, size, contents of cargo and functional effects [11, 159]. Conclusive information on functional modification was shown at Table 2.

**Table 2:** Technological advances towards functional modification for ASCs EVs

Modification	Strategy	Rationale	Ref
Engineering EVs^a^	Transfecting: mmu_circ_0000250, cirRNA_100395, miR-323-3p, miR-188-3p, miR-301a-3p, miR-29a-3p, miRR-28-3p, circAKap7, GDNF, miR-320d, miR‐375, miR-671, miR-191, miR-181-5p, miR-122, miR-21	Indirectly up-regulating expression of functional molecules into ASCs EVs	[14, 17, 21, 26, 34, 35, 44, 45, 49, 72, 77, 79, 82, 113, 125, 181]
Engineering EVs^b^	Transfecting: miR-381-3p, miR-10a, miR-21-5p	Directly up-regulating expression of functional molecules of ASCs EVs	[22, 37, 142]
Hypoxia	Hypoxia pre-condition in different methods	Enhancing pro-metabolism and pro-survival abilities. Angiogenesis, increasing levels of VEGF/VEGFR, attenuating inflammation. ECM repair/regeneration	[20, 67, 85, 86, 102, 103]
Controlled release	Biohybrid bovine bone matrix loaded with EVs	Continuous release of osteogenic factors for bone healing and regeneration	[50]
	polypeptide-based FHE hydrogel/oxygen releasing antioxidant and antibacterial cryogel wound dressing OxOBand/hyaluronic acid/polysaccharide-based dressing/alginate hydrogel loaded with EVs	Continuous release of EVs for diabetic/non-diabetic wound healing	[88, 139, 140, 144, 145]
Osteoinduction	ASCs were osteogenically induced using OM	Bone healing and regeneration	[104, 151, 182]
Chondrogenesis	ASCs were osteogenically induced using CM	Cartilage healing and regeneration	[160]
LLLI	A 24-h expose to LLLI before EVs collection	Reducing apoptosis of osteocyte induced by hypoxia	[152]
Inflammatory stimuli	TNF-α pre-condition for 3 days	Enhancing the potential of EVs in bone healing and regeneration	[114]
	IFNγ and TNFα	Increasing the immunosuppressive and anti-inflammatory potential of EVs	[92]
Growth factors	20 ng/ml PDGF, VEGF or FGF stimuli	Enhancing angiogenic potential	[19]
Lean adipose	Comparing ASCs EVs from lean and obese adipose	Having differences in size, cargo and bioactivities	[11, 159]

*ASCs EVs* adipose stem cells extracellular vesicles, *OM* osteogenic induction media, *CM* chondrogenic induction medium, *LLLI* low-level laser irradiation

^a^Indirectly modifying EVs by modifying functional molecules of ASCs

^b^Directly modifying functional molecules of ASCs EVs

### Systematic survey in aesthetic, plastic and reconstructive surgery

#### *Diabetic/non-diabetic wound healing (n* = *26)*

ASCs EVs delivered functional molecules for non-diabetic/diabetic wound healing via enhancing skin collagen production/angiogenesis/cell proliferation/migration/expression of wound healing-related growth factors, inhibiting apoptosis, promoting skin barrier function repair, reducing inflammation and scar formation, as well as regulating extracellular matrix remodeling [12, 14, 15, 32, 40, 51, 53, 73, 87, 88, 108, 119, 138–149, 166, 175]. The underlying mechanisms of action were shown as follows. For the capacity of promoting diabetic wound healing, ASCs EVs have been reported to regulate several axes such as mmu_circ_0000250/miR-128-3p/SIRT1 axis [14] in endothelial progenitor cell, miR-21-5p/Wnt/β-catenin signaling in keratinocytes [142], or transferring transcription factor nuclear factor-E2-related factor 2 (Nrf2) to endothelial progenitor cells [143]. The healing capability of ASCs EVs in non-diabetic wound involved the modulation of multiple signaling, such as the lncRNA H19/miR-19b/SOX9 axis in human skin fibroblast (HSF) cell [12], and miR-19b/CCL1/TGF-β pathway axis [40], AKT/HIF‑1α axis [53], Wnt/β‐catenin signaling [87], lncRNA MALAT1/miR-124/Wnt/β-catenin axis [141], miR-21/PI3K/AKT axis in HaCaT cells [148], as well as ERK/MAPK pathway in skin dermal fibroblasts [108]. The functional modification of ASCs EVs went through several processes from the simple to the complex. Initially, ASCs EVs without modification could be used topically or systemically. Then, a variety of regenerative biomaterials built up the concept of controlled EVs release, effectively matching with the complicated and long healing process of chronic wound [88, 139, 140, 144, 145]. Engineered EVs were another direction of achieving gene therapy by loading functional non-code RNA into the patent ASCs or EVs [14, 142].

#### *Other skin diseases and medical cosmetology (n* = *12)*

The pre-clinical studies indicated that ASCs EVs could promote epidermal barrier repair on the treatment of atopic dermatitis via increasing stratum corneum hydration, reducing the levels of multiple inflammatory cytokines, and enhancing de novo synthesis of ceramides [61, 97]. ASCs EVs could promote genes expression involved in skin barrier, lipid metabolism, cell cycle, and inflammatory response in the diseased area [61]. Only one study revealed that the intravenous injections of ASCs EVs could effectively slow-down the course of the systemic sclerosis via regulating miR-29a-3p/Dnmt3a/Pdgfrbb/Bcl2/Bcl-xl axis [35]. Two studies found that ASCs EVs could inhibit the proliferation/migration, and promote the apoptosis of keloid/hypertrophic scar fibroblasts via the regulation of miR-192-5p/IL-17RA/Smad axis [23] or inhibiting TGF-β1/Smad pathway [174]. Another two studies reported the essential roles of ASCs EVs in promoting the vascularization of skin flaps [163, 176], and one study found that ASCs EVs were comparable to parent ASCs in the inhibition of alloimmune response for vascularized composite allotransplantation [13]. Recently, ASCs EVs have been investigated in the antiaging of photoaged skin by increasing the mRNA expression of type I collagen, corresponding to the antiaging properties of parent ASCs [36].

Only three clinical studies have been reported in Korea for testing the therapeutic functions of hASCs EVs. Park et al. [16] tentatively applied ASCs EVs to the treatment of atopic dermatitis, and found that EVs could serve as an effective agent in the management of dupilumab facial redness. Two randomized controlled trials have indicated the safety and efficacy of hASCs EVs on the treatment of facial acne scars and skin brightening [136, 137].

#### *Angiogenesis/inflammation/fat grafts/hair regeneration (n* = *18)*

ASCs EVs could promote angiogenesis mainly via transferring functional microRNAs to targeted cells [19, 56, 85, 103, 122, 168, 169, 173, 177, 180, 181]. The underlying mechanisms for angiogenesis potential of ASCs EVs were shown as follows. Platelet-derived growth factor pre-conditioned ASCs EVs could load c-kit, SCF and matrix metalloproteinases that played a role in angiogenesis [19]. EVs derived from hypoxia-treated hADSCs showed angiogenesis capacity in fat grafting probably via regulating VEGF/VEGF-R signaling [85] and PKA signaling [103]. micro-RNAs derived from ASCs EVs also played an important role in angiogenesis. ASCs EVs could promote angiogenesis of endothelial cells by regulating miR-125a/DLL4 axis [122], miR-181b-5p/TRPM7 axis [173], miR-199-3p/sema3A axis [177] or miR-21/PTEN/AKT/ERK/HIF-1α/SDF-1 axis [181]. Xu et al. [169] found miR-423-5p from ASCs EVs mediated the proangiogenic activity of hADSCs by targeting Sufu. EVs isolated from Sirtuin 1 (SIRT1)-overexpressing ASCs unregulated Nrf2/CXCL12/CXCR7 signaling and promoted migration and tube formation of endothelial progenitor cells [180].

ASCs EVs also showed potential in attenuating inflammation and immune reactions probably via transferring functional molecules such as miR-34a, miR-124 and miR-135b [41, 84, 92]. Evidence has shown that ASCs could promote the survival rate of fat grafting via EVs secretion [27, 76, 85, 102]. Hao et al. [27] found that ASCs EVs could downregulate the level of transcription factor CCAAT/enhancer-binding protein via transferring let-7c. Corresponding to the poor angiogenesis/hypoxia in the early phase of fat grafting, the hypoxia-preconditioned ASCs EVs were superior to ASCs EVs in neovascularization and inflammation attenuation [102]. Recently, Wu et al. [183] indicted that that ASCs EVs could increase terminal hairs regeneration via promoting the expression of PDGF and VEGF in skin tissues.

#### *Cartilage and bone (n* = *19)*

A total of five studies investigated the functional roles of EVs from undifferentiated ASCs and chondrogenic ASCs in cartilage regeneration through modulating inflammation, promoting chondrocyte differentiation of ASCs, stimulating the migration/proliferation, and chondrogenic/osteogenic differentiation of BMSCs [29, 100, 105, 160, 161]. Zhao et al. [161] found that ADCs EVs could transfer miR‑145 and miR‑221 which could enhance cell proliferation and chondrogenic potential. In addition, proteomics analysis reveals that ASCs EVs could induce cartilage/bone regeneration probably by regulating signaling pathways including focal adhesion, ECM-receptor interaction, actin cytoskeleton, cAMP, and PI3K-Akt signaling pathways [29]. EVs LncRNA sequencing was also conducted to investigated the expression profile of lncRNAs, and several neighboring genes of differentially expressed lncRNAs that were involved in cartilage regenerations, such as TBX6, CHD4, and TRPV2 were identified [160].

A total of 14 studies have been published for investigating the functional effects on bone healing and tissue-engineered bone [50, 58, 63, 69, 75, 77, 90, 104, 114, 151, 152, 155, 156, 182]. ASCs EVs played an essential role of modulating functions of osteocytes and osteoclasts. Several studies have evidenced that ASCs EVs could be applied in the treatment of some bone damage-related pathologies such as diabetic osteoporosis, hypoxia/ischemia induced osteocyte apoptosis and osteocyte-mediated osteoclastogenesis [58, 90, 182]. The underlying mechanisms for these treatments could be attributed to inhibiting NLRP3 inflammasome activation in osteoclast [58], upregulating the radio of Bcl-2/Bax and diminishing the production of reactive oxygen species/cytochrome/caspase-9/caspase-3 [90]. Notably, Yang et al. [182] conducted EVs-miRNA sequencing in osteogenic differentiation of ADSCs and found some differentially expressed miRNAs connected osteogenic differentiation to processes such as axon guidance, MAPK signaling and Wnt signaling. In addition, ASCs could be pre-conditioned with tumor necrosis factor-alpha or low-level laser irradiation to mimic the inflammatory phase upon bone injury [114, 152].

Another essential role of ASCs EVs was to induce osteogenic differentiation, promote MSCs adhesion/migration/proliferation of MSCs via entrapping EVs on the surface of biohybrid bovine bone matrix [50], mineral-doped poly(L-lactide) acid scaffolds [63], or titanium [69]. The EVs proteome demonstrated that EVs carried proteins involving various integrins and integrin ligands, growth factors and growth factor receptors, as well as Wnts and MAPKs, which were related to adhesion, structure, morphology and GF activity [69]. In addition, Yang et al. [155] also found miR-130a-3p derived from ASCs EVs would regulate osteogenic differentiation of MSCs through mediating SIRT7/Wnt/β-catenin axis.

Engineered EVs s could also be designed specifically for osteogenic induction via altering expression of EVs-miRNAs. The simple methods were to induce the osteogenic differentiation of parent ASCs [104, 151, 182]. Other methods were directly loading specific miRNA such as miR‐375 into parent ASCs or EVs [77]. The EVs miR‐375 would inhibit insulin‐like growth factor binding protein 3 (IGFBP3) by binding to its 3′UTR and then improved the osteogenic differentiation of hBMSCs [77].

#### *Peripheral nerve injury (n* = *8)*

A total of eight studies have evidenced that ASCs EVs could exert therapeutic effects for peripheral nerve injury via increasing neurite outgrowth, improving neurotransmission function, modulating proliferation/migration/myelination of Schwann cells, and increasing secretion of neurotrophic factors [81, 96, 98, 124, 164, 165, 171, 172]. Notably, Ching et al. [165] found that Schwann cell-like phenotype-differentiated ASCs EVs contained mRNAs and miRNAs known to play a role in nerve regeneration. These EVs RNA could be transferred to neurons and promoted neurite outgrowth via down-regulating intrinsic inhibitors of regeneration.

## Discussion

The past decades have witnessed an upsurge in EVs research, primarily focusing on either disease markers or paracrine mediators for regenerative therapy. Every year, tremendous research funding is pouring into the preclinical and clinical studies of EVs. EVs even have been reported as potential regenerative cell-free medicine for *COVID-19* treatments [184, 185]. Indeed, obstacles have concurrently emerged when launching the clinical and industrial translation of EVs [186]. We give three hurdles urgently needing to be solved: poor follow to MISEV2018 guidelines, low yield, and unsatisfactory functional effects.

**Fig. 5:**
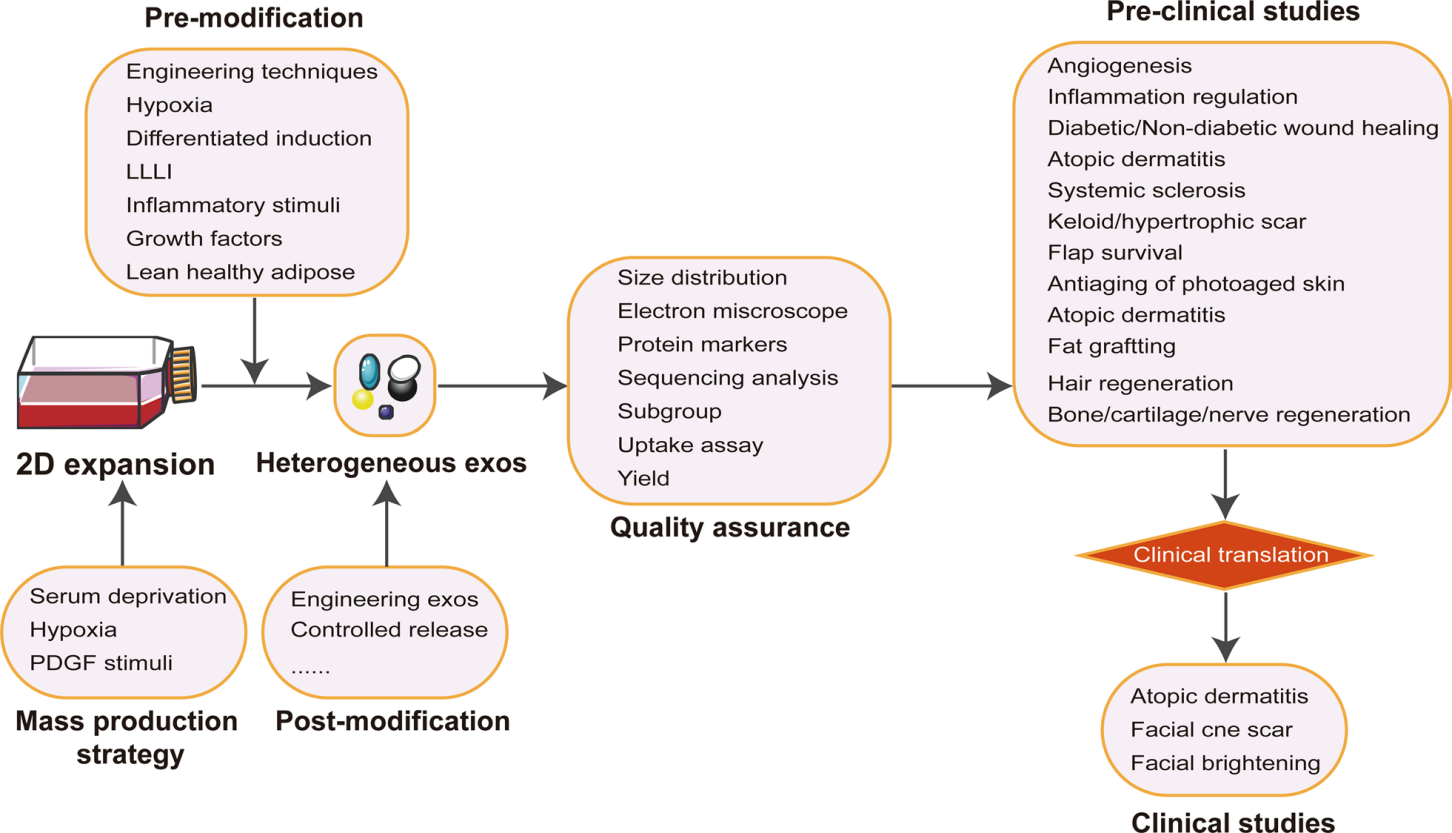
The recapitulative findings of our systematic survey

The findings are summarized at Fig. 5. Overall, the principal finding of our study was that the current studies were poorly recording minimal information for EVs study. First, some studies did not or unclearly disclose the species and cell types for EVs secretion. The functional effects of EVs depended largely on their parent cells. Notably, EVs form different types of MSCs such ASCs, BMSCs, umbilical cord blood MSCs (UCB-MSCs), and endometrium-derived MSCs (EnMSCs) could play different roles in tissue healing and regeneration [30, 115]. Secondly, we found that several minimal information for cell culture conditions, such as passage number, cell seeding density, and conditioned media collection time, were also poorly reported. These parameters could affect the yield or biological functions of ASCs EVs. Previous study indicated that both increasing frequency of collection and decreasing cell seeding density could increase EVs production, while the passage number beyond passage 4 was less effective in pro-vascularization bioactivity [187].

Additionally, culture medium components, such as basal medium, serum, growth factors, glucose and antibiotics, were the essential influence factors deserved special attention. However, there were still some studies not reporting the kinds of medium they used. The traditional culture for ASCs is the DMEM. However, most of them did not report the information of low-glucose or high-glucose. DMEM for ASCs culture easily caused low proliferation rate, early cell senescence and multi-lineage differentiation loss that were not helpful for mass production of ASCs EVs. DMEM/F-12, MEM α, specific MSC serum free medium and MSCM could be used for solving these obstacles. Using serum free medium or EVs-depleted serum could reduce the influence of serum-derived EVs to the functional assays. However, we found that there some of studies using serum without EVs depletion. Overall, we highlight the necessity for careful consideration of cell culture parameters.

Almost all studies stored the conditioned media at − 80 °C, or firstly isolated EVs and then stored it at − 80 °C. Whether the long-term cryopreserved EVs is different from those freshly isolated in terms of morphology and function deserves special attention. ASCs EVs freeze-dried powder may be safe and a long-term storage alternative. After rehydration, ASCs EVs were still stable in the membrane morphology and components.

The obstacles to large-scale production and clinical translation of ASCs EVs are the inefficient isolation techniques along with the high costs and low purification. Ultracentrifugation still remains the gold standard to concentrate EVs, despite the defects of low yield and time consumption. The EVs isolation kit only could be used to isolate EVs from little conditioned medium. Ultrafiltration combined with ultracentrifugation could be translated to large-scale EVs manufacturing. Recently, the tangential flow filtration (TFF) and size exclusion chromatography (SEC) have been proposed as an effective concentration methods for large volumes of conditioned media [186]. Another strategy for large-scale manufacturing is to increase the number of secretion by stimulating ASCs. In our study, we found several methods for optimal production of ASCs EVs, such as hypoxia pre-condition, PDGF pre-condition and serum starvation.

Next, EVs identification via several complementary techniques, such as TEM, NTA and protein markers, is essential to quality control of EVs. However, there was still 4.02% of studies not reporting any identification methods. Only 56.90% of studies reported the size distribution while 81.61% of studies provided images of single EVs at high resolution. In addition, there were 82.18% of studies evaluated the protein markers mainly involving those transmembrane/lipid-bound protein and cytosolic protein. Actually, a study by Mathieu et al. [188] has evidenced that exosomes might specifically bear CD63 combined with some late endosome proteins but little CD9. Notably, our study found that there was 18.39% of publication not reporting the quantification of EVs. The BCA for total proteins yield was most used to reported EVs quantification.

Some preclinical and clinical studies were included in our systematic survey, involving one case series [16] and two randomized controlled trials [136, 137]. Overall, the current articles have given some therapeutic evidence for the functional roles of ASCs EVs in aesthetic, plastic and reconstructive surgery. In our study, we found three kinds of strategies could be used for optimizing the functional roles of ASCs EVs: engineering EVs, targeted precondition of parent ASCs and controlled EVs release.

We found several obstacles to the promotion of EVs research. Firstly, the functional roles were attributed to uptake of ASCs EVs by receipt cells rather than soluble non- EVs associated mediators from conditioned media. This was especially right when isolating EVs from polymer-based concentration kits. However, in our study, we found 62.07% of included studies did not reported any assays related to functional uptake. Besides, we found that there were 36.21% of included studies not reporting the working concentration. The clear reporting of working concentration undoubtedly increased the reliability and reproducibility of published results.

## Conclusion

Our study highlights a normative reporting for EVs research, referring to MISEV2018 guidelines to increase robustness of results. Technological advances towards mass production and functional modification should be further improved for the translation of clinical practices and industrial manufacturing.

## Data Availability

Not applicable.
